# Peptide Occurring in Enterobacteriaceae Triggers *Streptococcus pneumoniae* Cell Death

**DOI:** 10.3389/fcimb.2019.00320

**Published:** 2019-09-10

**Authors:** Fauzy Nasher, Min Jung Kwun, Nicholas J. Croucher, Manfred Heller, Lucy J. Hathaway

**Affiliations:** ^1^Institute for Infectious Diseases, Faculty of Medicine, University of Bern, Bern, Switzerland; ^2^Graduate School for Cellular and Biomedical Sciences, University of Bern, Bern, Switzerland; ^3^Department of Infectious Disease Epidemiology, MRC Centre for Global Infectious Disease Analysis, Imperial College London, London, United Kingdom; ^4^Proteomics and Mass Spectrometry Core Facility, Department for BioMedical Research, University of Bern, Bern, Switzerland

**Keywords:** *Streptococcus pneumoniae*, peptide, non-encapsulated, aliB-like ORF 1, transcriptome, proteome, cell death

## Abstract

Non-encapsulated S*treptococcus pneumoniae* often possess two genes, *aliB-*like ORF 1 and *aliB-*like ORF 2, in place of capsule genes. *AliB*-like ORF 1 is thought to encode a substrate binding protein of an ABC transporter which binds peptide SETTFGRDFN, found in 50S ribosomal subunit protein L4 of Enterobacteriaceae. Here, we investigated the effect of binding of AliB-like ORF 1 peptide on the transcriptome and proteome of non-encapsulated pneumococci. We found upregulation of gene expression of a metacaspase and a gene encoding N-acetylmuramoyl-L-alanine amidase, both of which are proposed to be involved in programmed cell death in prokaryotic cells. Proteome profiling indicated upregulation of transcriptional regulators and downregulation of metabolism-associated genes. Exposure to the peptide specifically triggered death in pneumococci which express AliB-like ORF 1, with the bacteria having an apoptotic appearance by electron microscopy. We propose that binding of the AliB-like ORF 1 peptide ligand by the pneumococcus signals a challenging environment with hostile bacterial species leading to death of a proportion of the pneumococcal population.

## Introduction

*Streptococcus pneumoniae (S. pneumoniae)* (pneumococcus) is a common asymptomatic colonizer of the human nasopharynx. The human nasopharynx is an ecosystem that hosts a diverse microbial community and we have previously proposed that pneumococcus senses bacteria in the environment through short peptide fragments derived from these other bacterial species sharing the same niche (Hathaway et al., 2014; Nasher et al., 2018a,b).

The multilocus sequence types (MLST) 344 and 448 consist of non-encapsulated pneumococci and make up a phylogenetic group that clusters away from the majority of encapsulated pneumococci (Hathaway et al., 2004; Hanage et al., 2006; Hilty et al., 2014). Non-encapsulated pneumococci make up 10% of those isolated from the nasopharynx (Finland and Barnes, 1977; Carvalho et al., 2003) and in a global study we found that 88% of non-encapsulated pneumococci of the classic lineage belonged to either MLST 344 or 448 (Hilty et al., 2014). These non-encapsulated pneumococci have two genes in place of the capsule genes that we named *aliB-*like ORF 1 and *aliB-*like ORF 2 due to their 61–64.5% homology to the ATP-binding cassette (ABC) transporter substrate binding protein gene *aliB* (and 62% homology to each other) (Hathaway et al., 2004). We predicted that *aliB-*like ORFs encode substrate-binding proteins due to their homology to *aliB* which is found in all pneumococci (Hathaway et al., 2004; Hilty et al., 2014).

AliB-like ORF1 binds to peptide of sequence SETTFGRDFN, which is found in the 50S ribosomal subunit protein L4 of Enterobacteriaceae including *Klebsiella pneumoniae* (Hathaway et al., 2014) (i.e., binds to totally different peptide than AliB whose ligand is AIQSEKARKHN, matching 30S ribosomal protein S20) (Nasher et al., 2018b). Secreted proteins of *Escherichia coli (E. coli)* include ribosomal associated proteins of the 50S ribosomal subunit (Nasher et al., 2018b). Incubation with *E. coli* reduces the number of live pneumococci which have the AliB-like ORF 1 receptor but not those which lack the receptor (Hathaway et al., 2014). In the presence of competence stimulating peptide (CSP), binding of AliB-like ORF 1 to its ligand increases competence for genetic transformation. In contrast, AliB-like ORF 2 peptide ligand, FPPQSV, is found in proteins of *Prevotella* species (Hathaway et al., 2014) and binding of AliB-like ORF 2 to its peptide ligand leads to increased carbohydrate metabolism and increased growth (Nasher et al., 2018a).

Here, we investigated the effect of binding of AliB-like ORF 1 to its peptide ligand, SETTFGRDFN, on changes to the pneumococcal transcriptome, proteome, growth, and survival in chemically defined medium (CDM). Understanding the interactions that occur between pneumococci and other bacterial species in nature may indicate targets for therapeutic interventions.

## Materials and Methods

### Bacterial Strains and Culture

The Swiss non-encapsulated nasopharyngeal pneumococcal isolate 110.58 of multilocus sequence type (MLST) ST344, and its mutant in which *aliB-*like ORF1 has been inactivated (mutant ΔORF 1) have been described previously (Hathaway et al., 2004, 2014; Hilty et al., 2014). The bacteria were stored at −80°C using Protect bacterial preservers (Technical Service Consultants, Heywood, UK), were grown on CSBA plates at 37°C, 5% CO_2_. 5–10 colonies in 5 ml brain heart infusion (BHI) broth (Becton Dickinson and Company, le Pont de Claix, France) containing 5% fetal calf serum (FCS; Biochrom KG, Berlin, Germany) (BHI + FCS) were used to prepare overnight cultures.

### Gene Expression Analysis by RNA-Seq and Real-Time RT-PCR

RNA extraction was performed as described previously (Nasher et al., 2018a), for strains 110.58 and its mutant ΔORF 1. The bacteria were plated out on CSBA plates, incubated at 37°C, 5% CO_2_ overnight, colonies picked and cultured in 5 ml BHI + FCS overnight until OD_600nm_ = 0.4 then centrifuged at 2000 g for 5 min and the pellet washed with 5 ml CDM (chemically defined medium that contains no peptides) and centrifuged at 200 g for 5 min. The pellet was resuspended in 5 ml CDM and 500 μl of this bacterial suspension was added to 9.5 ml CDM to make a final volume of 10 ml. For each strain, at exactly OD_600nm_ = 0.2 the culture was split into two, each tube containing 5 ml. To one tube for each strain, a final concentration of 0.07 mg/ml of the peptide SETTFGDRFN (synthesized by PolyPeptide Group, Strasbourg, France) was added and all tubes incubated at 37°C for exactly 15 min. Transcription was stopped by adding RNAprotect (Qiagen) and RNA extracted as described previously (Nasher et al., 2018a). RNA was acquired from three independent experiments performed on three separate days for RNA-Seq and a further three independent experiments for real-time RT-PCR.

For RNA-Seq, ribosomal RNA was depleted using Ribominus (Invitrogen) and RNA purification was carried out with RNA Clean & Concentration™-5 kit (Zymo Research) following the manufacturers' instructions and purified RNA was eluted in 6 μl. TruSeq® Stranded mRNA (Illumina) was used to prepare libraries, 13 μl of Fragment, Prime, Finish Mix was added to 5 μl of the mRNA. Fragmentation was performed for 40 s at 94°C. An Illumina 3000 Hi-Seq paired end sequencing platform was used to acquire raw reads.

Analysis was carried out as follows: the reads for the forward and reverse ends were paired and mapped on to the reference strain*, S. pneumoniae strain* NT_110_58 (Accession number-NZ_CP007593), using Bowtie2 (version 2.3.4.1) (Hathaway et al., 2014) with the overall alignment rates above 99%. The range of sequenced fragments was 35–151, the mean length of sequenced fragments was 144 and the sequencing and coverage was on 2503 times the genomic size (ranging between 883 and 4,074). Prior to the mapping step, the reads were trimmed and filtered using Trimmomatic (version 0.36) (Bolger et al., 2014). Cuffdiff (version 2.2.1) RNA-Seq analysis tool was used to compare expressions levels of genes and it used 5% for false discovery rate for the analysis as described previously (Trapnell et al., 2013).

Expression of genes of interest identified by RNA-Seq was quantified by real-time RT-PCR as described previously (Hathaway et al., 2007) and normalized against 16S. Primers and probes are shown in Table 1.

**Table 1:** Primers and probes used in real-time RT-PCR.

**Gene**	**Forward primer**	**Reverse primer**	**Probe (all with 6-FAM 5^**′**^ and MGB 3^**′**^)**
16S	GACGATACATAGCCGACCTGAGA	GTAGGAGTCTGGGCCGTGTCT	CCAGTGTGGCCGATC
*lytFN1 (SpnNT_01010)*	GGACGCCAATTCCCAACCA	ATGTCATGGAGGAAGAGATGGAAGAA	AATGGCCTAGCATTTTC
*lytFN2 (SpnNT_01011)*	CAACGCTTCCAACTGTAACATCAG	GCCGAACAATTCTTCCTGGTTTAAT	CTTCACGCCAAGCAAC
*pnuC*	CGACAAACTCCTGCTTTTCCTTCT	TGCAGCCAATTGGACTTCTAGTT	CTGTGCCTGATAAATC
*mtrR*	GGCCAATTGAATTACTCTAGCTTCCT	CAGACCTTTATTTGGTTTGGGTTCAC	CTGGCCTCCCGGAAAA
*clpC_1*	ATCCTGTAACAGATGTGCATGCT	CTCAACAGCTTTTTCTTGCTTGTCT	TTTCTGCCTCAATTTC
*ccpA*	TGTAGATAAGCCATAAATGACTTTT-	TGGTTGCTTATGGGAGGAGAATC	ACGCACACCTCTTTCA
	AAATCCATAAAGTAAT		

### Proteomic Analysis

Bacterial strain 110.58 and its mutant ΔORF 1 were plated out on CSBA plates, incubated at 37°C, 5% CO_2_ overnight, colonies picked and cultured in 5 ml BHI + FCS overnight until OD_600nm_ = 0.4 then centrifuged at 2,000 g for 5 min and the pellet washed with 5 ml CDM and centrifuged at 200 g for 5 min. The pellet was resuspended in 5 ml CDM and 500 μl of this bacterial suspension was added to 9.5 ml CDM to make a final volume of 10 ml. For each strain, at exactly OD_600nm_ = 0.2 the culture was split into two, each tube containing 5 ml. To one tube for each strain, a final concentration of 0.07 mg/ml of the peptide SETTFGDRFN (synthesized by PolyPeptide Group, Strasbourg, France) was added and all tubes incubated at 37°C for exactly 15 min. Sample processing, LC-MS/MS and data interpretation was essentially done as described previously (Nasher et al., 2018a). Briefly, LC-MS/MS analysis was carried out on an Ultimate3000 nanoLC coupled to an Orbitrap Fusion Lumos instrument (ThermoFisher Scientific). Full MS scans in the range of 400–1,400 m/z were acquired in the orbitrap at 120,000 resolution with AGC set to 4e5 and maximal ion injection time of 50 ms. Peptide precursors with charge 2–8 were fragmented once in the ion trap then excluded for 30 s. The ion trap setting were data-dependent MS2 cycle time of 3 s, isolation width of 1.6 m/z, fragmentation HCD mode with 30% normalized collision energy, AGC of 1e4 with maximal ion injection time of 35 ms. The LC-MS/MS data was processed with MaxQuant (version 1.5.4.1) using default settings for peak detection, strict trypsin cleavage rule was used and up to three missed cleavages were allowed, variable oxidation on methionine and acetylation of protein N-termini with strict carbamidomethylation of cysteines. A retention time window of 0.7 min was activated between runs. The fragment spectra were interpreted with the *S. pneumoniae* ensemble database (version gca_000817005_ASM81700v1). The normalized label-free protein intensities (LFQ) as calculated by the MaxQuant software were submitted for a differential protein expression analysis. For this, missing LFQ values were imputed from the low end of the LOG2 transformed intensity distribution of each LC-MS/MS run, when there was at least one valid LFQ value within the six replicates of each sample. Otherwise, missing values were replaced by zero as suggested by Lazar et al. (2016). Differential protein expression analysis was performed with *t*-tests within Perseus (version 1.5.5.3), including a permutation-based false discovery rate estimation to correct for multiple testing. Differential expression was accepted with a 5% false discovery rate and a minimal LOG2-fold-change of two by applying the S0 function (set at 0.5).

### Growth Measurement

Following overnight growth on CSBA plates at 37°C, 5% CO_2_, the bacteria were sub-cultured in BHI + FCS and grown to OD_600nm_ 0.5 and then centrifuged at 3,000 g for 5 min. The bacteria were resuspended in CDM containing 5.5 mM glucose (Schaffner et al., 2014) to give a starting OD_450nm_ of 0.01. The growth was monitored as described previously (Hathaway et al., 2012) in sterile flat-bottomed 96-well microtitre plates (Nunclon Surface, Nunc, Denmark) based on the method of Brewster (Brewster, 2003); 200 μl bacteria culture was incubated per well at 37°C and OD_450nm_ was measured every 30 min by a VERSAmax microplate reader (Molecular Devices) for 22 h with 5 s of automatic shaking preceding each reading. Condensation was prevented by pre-treating the plate lids with 3 ml 0.05% Triton X-100 in 20% ethanol (Hathaway et al., 2012).

### Growth Competition Assay

Wild type strain 110.58 and mutant ΔORF 1 were streaked onto CSBA plates and incubated at 37°C in a 5% CO_2_-enriched atmosphere overnight then sub-cultured in 5 ml BHI + FCS medium to OD_600nm_ 0.5, centrifuged at 3000 g for 5 min and resuspended in 5 ml CDM. Two hundred fifty microliter of each bacterial culture was transferred to 4.5 ml CDM, pre-warmed to 37°C, with and without AliB-like ORF 1 ligand SETTFGRDFN at a final concentration of 0.07 mg/ml and the culture incubated to OD_600nm_ 0.3. Serial dilutions in PBS were plated onto CSBA plates with and without 3 μg/ml chloramphenicol to select between the wild type (chloramphenicol susceptible) strain and the ΔORF 1 mutant (resistant). After overnight incubation, the number of colonies was counted and the colony forming units (CFU) for each strain calculated. For the wildtype, CFU was calculated by subtracting the number of colonies on the chloramphenicol plates from the number of colonies on CSBA.

### Enumeration of Live/Dead Cells by Microscopy

The effect of the ORF 1 peptide was also observed microscopically using LIVE/DEAD™ BacLight™ assay kit (Thermo Fisher Scientific). *Streptococcus pneumoniae* strains 110.58 and mutant ΔORF 1 were cultured in 5 ml CDM until exponential growth phase, ORF 1 peptide was added to a final concentration of 0.07 mg/ml and incubated at 37°C for 30 min. The cells were centrifuged at 3,000x g for 10 min, resuspended in sterile phosphate-buffered saline (PBS) and stained with LIVE/DEAD™ *Bac*Light™ bacterial viability kit as per manufacturer's instructions. The bacteria suspension was pipetted onto a microscope slide and a coverslip applied firmly. The slides were viewed using a Zeiss Axio Imager M1 fluorescence microscope with a 20 X objective and photographed by a Zeiss AxioCam HRc camera.

For quantification of green (live) and red (dead) cells, the images were analyzed with ImageJ software (Rueden et al., 2017). Each image contained between 96 and 620 bacteria, five images were counted per group in each of three independent experiments making a total of 15 images per group. Each data point in **Figure 3B** represents one image.

### Electron Microscopy

*Streptococcus pneumoniae* strains 110.58 and mutant ΔORF 1 were cultured in 5 ml CDM until exponential growth phase, ORF 1 peptide was added to a final concentration of 0.07 mg/ml and incubated at 37°C for 30 min. The bacteria were pelleted by centrifugation (5,000 rpm for 5 min) and the supernatant was discarded. Bacteria were then cryopreserved by high-pressure freezing (HPF) as described in Studer et al. (2001) using 1.4 x 0.1 mm membrane carriers (Leica Microsystems, Vienna) coated with L-α-phosphatidylcholine (Fluka, Buchs, Switzerland) (Studer et al., 2001). Acetone containing 2 % osmium tetroxide, 0.1% uranyl acetate, 0.2% ruthenium hexamine trichloride (RHT) and a total of 4% H_2_O served as medium for freeze substitution (FS). After substitution bacteria were washed in acetone (Merck, Darmstadt, Germany) for four times 30 min, they stayed then for 2 h 30 min in acetone-Epon (2:1) and 4 h in acetone -Epon 1:1 followed by an overnight incubation with acetone -Epon 1:2 at room temperature. The next day, samples were embedded in Epon (Sigma-Aldrich, Buchs, Switzerland) and left to harden at 60°C for 5 days. Sections were produced with an ultramicrotome UC6 (Leica Microsystems, Vienna, Austria), first semithin sections (1 μm) for light microscopy which were stained with a solution of 0.5% toluidine blue O (Merck, Darmstadt, Germany) and then ultrathin sections (75 nm) for electron microscopy. The sections, mounted on Formvar® (Ted Pella Inc. USA) coated single slot copper grids, were stained with Uranyless (Electron Microscopy Sciences, Hatfield, USA) and lead citrate (Leica Microsystems, Vienna, Austria) with an ultrostainer (Leica Microsystems, Vienna, Austria). Sections were then examined with a transmission electron microscope (CM12, Philips, Eindhoven) equipped with a digital camera (Morada, Soft Imaging System, Münster, Germany) and image analysis software (iTEM).

### Statistical Analysis

Unless otherwise stated, student *t-*tests were performed to obtain *p*-values using the software GraphPad Prism (Version 7, GraphPad Software, Inc.).

## Results

### AliB-Like ORF1 Peptide Affects Expression of Genes Involved in Cell Death and Metabolism

To identify the key regulatory changes triggered by the signaling through ORF1 in response to its ligand, RNA-Seq was used to identify differentially expressed genes in strain 110.58 and its ΔORF 1 mutant in the presence and absence of peptide SETTFGRDFN. The inherent difference in gene expression between the 110.58 and its ΔORF 1 mutant in the absence of the peptide is shown in Table S1. Exposure to the ORF 1 ligand, SETTFGRDFN, for 15 min caused changes in gene expression in both the 110.58 and in the ΔORF 1 mutant. In the mutant, 7 genes were significantly upregulated at least 2-fold in the presence of the peptide: *metF, metE, pyrP, nanB carA, patB_2*, and *paaI* with roles in carbon, pyrimidine, amino acid and fatty acid metabolism. Twenty one genes were downregulated at least 2-fold in the mutant plus peptide including those involved in purine biosynthesis (*purC, purL, purM*). Twenty six genes were significantly upregulated and 50 downregulated in total, see Table S2 for expression of all genes. However, a different pattern of expression was seen in the wild type strain: Genes that were significantly upregulated or downregulated in the wild type following exposure to the ORF 1 peptide ligand are shown in Table 2. Tables with all the genes affected by the peptide in the wild type strain are shown in Table S3.

**Table 2:** Significantly upregulated and downregulated genes in the wild type pneumococcal strain 110.58 following exposure to 0.07 mg/ml of ORF 1 peptide ligand SETTFGRDFN.

	**Gene number**	**Gene name**	**Description of product**	**Ratio^a^**
**UPREGULATED**				
	***SpnNT_01010***	***lytFN1***	**Caspase superfamily domain protein, peptidase C14**	**5.4**
	***SpnNT_01011***	***lytFN2***	**N-acetylmuramoyl-L-alanine amidase family protein**	**3.8**
	*SpnNT_02122*		hypothetical protein	2.7
	*SpnNT_01883*	*dpnM*	Modification methylase DpnIIA	2.6
	***SpnNT_01860***	***ccpA_1***	**Catabolite control protein A**	**2.4**
**DOWNREGULATED**				
	***SpnNT_01892***	***pnuC***	**Nicotinamide riboside transporter**	**3.1**
	*SpnNT_00711*	*pacS*	Cation-transporting ATPase	3.0
	*SpnNT_00097*	*catE*	Catechol-2,3-dioxygenase	2.9
	***SpnNT_00332***	***clpC*****_1**	**putative ATP-dependent protease subunit**	**2.8**

a *Ratio is the fold difference of the gene in the presence of peptide SETTFGRDFN compared to in its absence. Genes hightlighted in bold were chosen for RT-PCR which confirmed the RNA-Seq findings*.

SETTFGRDFN peptide caused significant upregulation of 5 genes in the wild type strain but not the mutant; the two most upregulated genes, which we have named *lytFN1* and *lytFN2*, encode a caspase superfamily domain protein peptidase C14 and an N-acetylmuramoyl-L-alanine amidase family protein respectively, and are expected to degrade cell wall peptidoglycan and play a role during cell death. However, it must be noted that expression of *lytFN1* and *lytFN2* also differed between the wildtype and its mutant in the absence of peptide, for as yet unknown reasons. Other upregulated genes included *dpnM*, which encodes DNA modification methylase and *ccpA*, a catabolite control protein.

Four genes were significantly downregulated in the wild type strain 110.58, but not the mutant, in the presence of the peptide: *pnuC*, encoding a transporter involved in nicotinamide adenine dinucleotide biosynthesis, *pacS* encoding a metal ion transporter component, *catE* encoding a catechol-2,3-dioxygenase, and *clpC* encoding an ATP-dependent protease subunit. These were all the significant changes in the wild type induced when the peptide was added. The genes highlighted in bold in Table 2 were chosen for RT-PCR and confirmed the RNA-Seq findings (Figure S1).

### The Proteomic Profile Indicates Peptide Induces Transcriptional Regulators and Downregulates Metabolism

To determine whether changes seen in gene transcription are translated into changes at the protein level, proteomic analysis was performed by LC-MS/MS on wild type strain 110.58 and mutant ΔORF 1 with and without exposure to AliB-like ORF 1 peptide ligand SETTFGRDFN. Table 3 shows all proteins for which expression was significantly upregulated or downregulated in 110.58 following exposure to ORF 1 peptide for 15 min. (All the proteins affected by the peptide in the wild type strain and its ΔORF 1 mutant are shown in Table S4. Table S5 shows the proteomic profile of the wild type and mutant in the absence of peptide; Table S6 the mutant with and without the peptide and Table S7 the wild type with and without the peptide). LytFN1 and LytFN2 did not appear in the significantly upregulated proteins which we speculate is because the timepoint is too early for their transcripts to have been translated into protein. The protein PadR family transcriptional regulator, associated with stress response in *S. pneumoniae* (Liu et al., 2017), was significantly upregulated. Furthermore, amino acid synthesis associated repressor protein GlnR and a putative efflux pump repressor MtrR were also upregulated. Proteins involved in sugar uptake and carbohydrate metabolism were downregulated, such as GmuD_1, a methyl beta-D-glucoside-6-phosphate glucohydrolase, LicC_2, a phosphotransferase system (PTS) lichenan-specific EIIC component. Transcriptional regulators and metabolism associated proteins are highlighted in bold in Table 3.

**Table 3:** Upregulated and downregulated proteins in the wildtype strain 110.58 in the presence of 0.07 mg/ml of ORF 1 peptide ligand SETTFGRDFN.

	**Protein number**	**Protein name**	**Function**	**Ratio^a^**	**q**
**UPREGULATED**					
	**AJD71059**	**PadR family transcriptional regulator**	**Lineage-specific thermal regulator protein**	**Infinite**^b^	**0**
	AJD72039	XseB	Exodeoxyribonuclease VII activity	11.1	0
	AJD71512	VanYB	Serine-type D-Ala-D-Ala carboxypeptidase activity	4.6	0
	AJD71029	SorB_1	Sorbose-specific phosphotransferase enzyme	4.6	0.007
	**AJD71397**	**GlnR**	**Glutamine/glutamate synthetase repressor**	**1.7**	**0.008**
	**AJD71668**	**MtrR**	**Transcriptional regulator**	**1.8**	**0.008**
	AJD73064	RlmA	23S rRNA (guanine(748)-N(1))-methyltransferase activity	1.5	0.009
**DOWNREGULATED**					
	AJD71629	PyrF_1	Catalyzes the decarboxylation of orotidine 5'-monophosphate (OMP) to uridine 5'-monophosphate (UMP). (de novo Pyrimidine biosynthesis)	Infinite^b^	0
	**AJD71208**	**GmuD_1**	**Methyl beta-D-glucoside-6-phosphate glucohydrolase activity (carbohydrate metabolic process)**	**Infinite**^b^	**0**
	AJD71067	SdhA	Iron-binding, L-serine ammonia-lyase activity	11.3	0
	AJD71445	HHE	Hemerythrin cation binding domain protein	5.8	0.001
	**AJD72972**	**LicC_2**	**Phosphotransferase system (PTS) lichenan-specific EIIC component**	**3.4**	**0.002**
	AJD72365	IscS_2	Cysteine desulfurase activity	3.4	0.003
	AJD71048	SaeS	Histidine kinase phosphorelay sensor kinase activity	3.3	0.005
	AJD72932	RmuC	Recombinase	3.2	0.006
	AJD71129	CorA	metal ion transmembrane transporter activity	2.8	0.006
	AJD72642	macB_1	Putative ABC transporter ATP-binding protein; part of the ABC transporter complex macAB involved in macrolide export.	2.7	0.006
	AJD71758	YbiV	Sugar phosphatase	2.3	0.008

a *Ratio is the fold difference of the protein in the presence of ORF 1 peptide compared to in its absence*.

b *The value in the absence of peptide was 0 so fold change in the presence of peptide could not be calculated*.

*Transcriptional regulators and metabolism associated proteins are highlighted in bold*.

### ORF 1 Peptide Ligand Causes Cell Death in the Wild Type Strain 110.58

**Figure 1:**
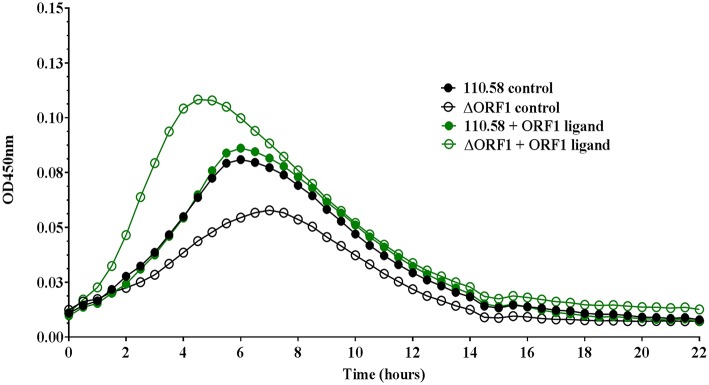
Growth analysis of pneumococcal strain 110.58 and its mutant Δ ORF 1 in the presence and absence of 0.062 mg/ml of ORF 1 peptide. Curves show the mean optical density values over time of three independent experiments performed on different days.

Growth in CDM was determined by measuring optical density (OD) over time for wild type strain 110.58 in the absence (control) and presence of AliB-like ORF 1 peptide ligand SETTFGRDFN. In the absence of the peptide, wild type strain 110.58 had a growth advantage over its mutant ΔORF 1 (Figure 1). The peptide boosted growth of the ΔORF 1 mutant but not the wild type strain. Growth curves were performed with 4 different doses of ORF 1 peptide ligand in each of three independent experiments on three different days. The mean values for all doses, with error bars showing standard error, are shown in Figure S2.

We also tested the effect of the ORF 1 peptide on the number of live bacteria by plating out and counting CFU at different time points during exponential growth (Figure S3). This indicated a slight (non-significant) reduction in live bacteria for the wild type 110.58, but a significant increase in number of live bacteria for the ΔORF 1 mutant, in line with the growth curve results in Figure 1.

**Figure 2:**
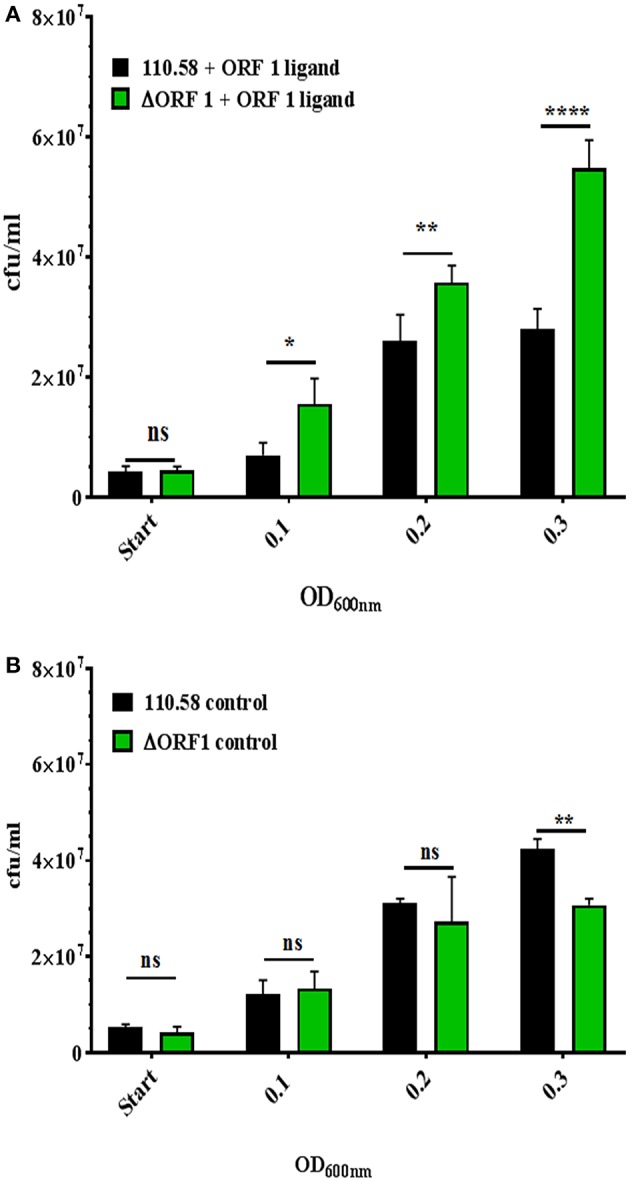
Competition assay between strain 110.58 and its mutant ΔORF 1 when cultured together in CDM. Strain 110.58 was cultured together with its mutant ΔORF 1 **(A)** in the presence (0.07 mg/ml) or **(B)** absence of ORF 1 peptide. When OD_600nm_ reached 0.1, 0.2 and 0.3, the bacteria were plated onto CSBA with and without 3 μg/ml of chloramphenicol to differentiate between the wild type and the mutant and cfu counted. Results are presented as mean values ± s.d. of three independent experiments. ^*^p = 0.0032; ^**^p = 0.0021; ^****^p < 0.0001; ns, not significant.

Given the findings on growth, next we compared 110.58 and ΔORF 1 mutant in competition assay in the presence and absence of ORF 1 peptide. Figure 2A shows that 110.58 is outcompeted by the mutant lacking AliB-like ORF 1 (ΔORF 1) when the ORF 1 peptide is present. The wild type strain 110.58 outcompetes its ΔORF 1 mutant when the ORF 1 peptide is absent (Figure 2B), in agreement with the growth curve results of Figure 1.

**Figure 3:**
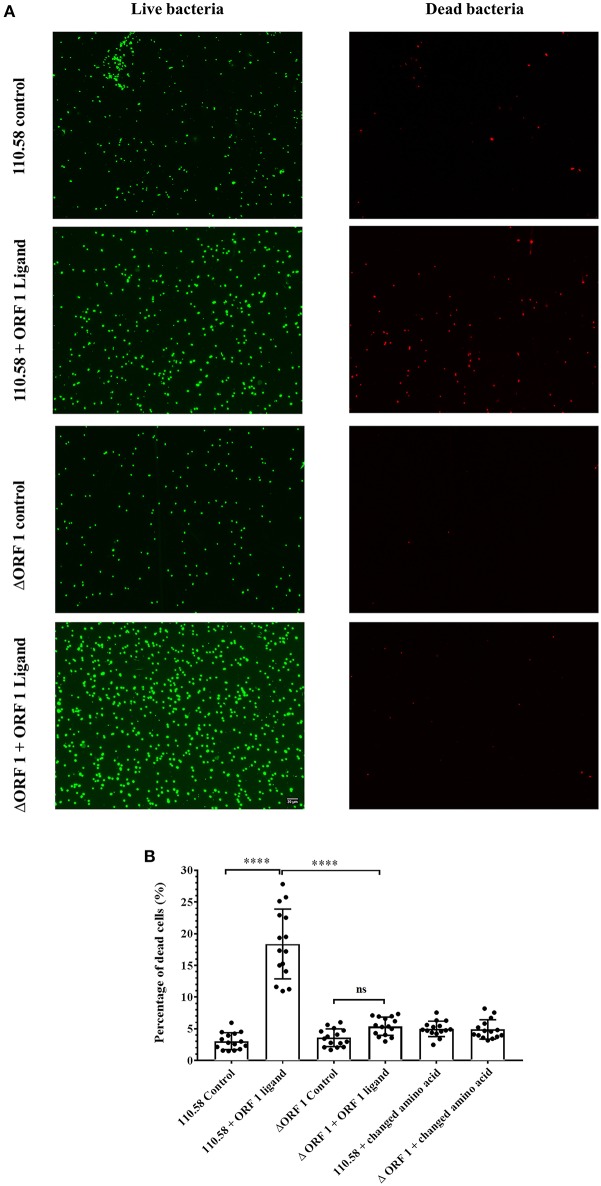
Results of LIVE/DEAD™ *Bac*Light™ bacterial viability visualized by fluorescence microscopy. **(A)** *Streptococcus pneumoniae* strain 110.58 and ΔORF 1 mutant in the presence (0.07 mg/ml) or absence of the ORF 1 peptide. Damaged membrane causes dead bacteria to appear red whereas live cells with an intact membrane appear green. All panels are at the same magnification. **(B)** Quantification of the red cells presented as percentages. “Changed amino acid” refers to use of a peptide with a single amino acid substitution (SETTFGR**E**FN) instead of ORF 1 peptide (SETTFGR**D**FN). Results are shown as the mean ± s.d. of three independent experiments ^****^p < 0.0001; ns, not significant.

Given the outcome of the growth and competition assays and the finding that a caspase family gene is upregulated by the ORF 1 peptide (although we have not investigated the role further by making a knockout), we investigated the effect of ORF 1 peptide on cell death. Strain 110.58 wildtype and its mutant were incubated with the ORF 1 ligand for 30 min in the presence of fluorescent dyes STYO 9 and propidium iodide. Bacteria cells were visualized using fluorescent microscopy. *Streptococcus pneumoniae* cells with damaged membrane appear red and are considered dead, whereas cells with an intact membrane appear green. Figure 3A shows that ORF 1 peptide increased the number of dead (red) bacteria for the wild type strain 110.58 but not the ΔORF 1 mutant. There were more live (green) bacteria in the ΔORF 1 mutant treated with ORF 1 peptide than any of the other groups as expected since the ORF 1 peptide increases growth of the ΔORF 1 mutant. The percentage of dead cells was quantified (Figure 3B). This confirmed that ORF 1 peptide caused a significant increase in the percentage of dead cells only for the wild type 110.58 strain. The effect was specific to the ORF 1 peptide SETTFGRDFN as the effect was not seen when peptide with a single amino acid substitution (SETTFGREFN) was used instead. The increase in number of live wild type bacteria stimulated by the ORF 1 peptide approximately equals the amount of cell death that the peptide causes. The consequence of this is that the number of live bacteria remains constant, in agreement with the data enumerating live CFU at exponential growth phase in Figure S3.

**Figure 4:**
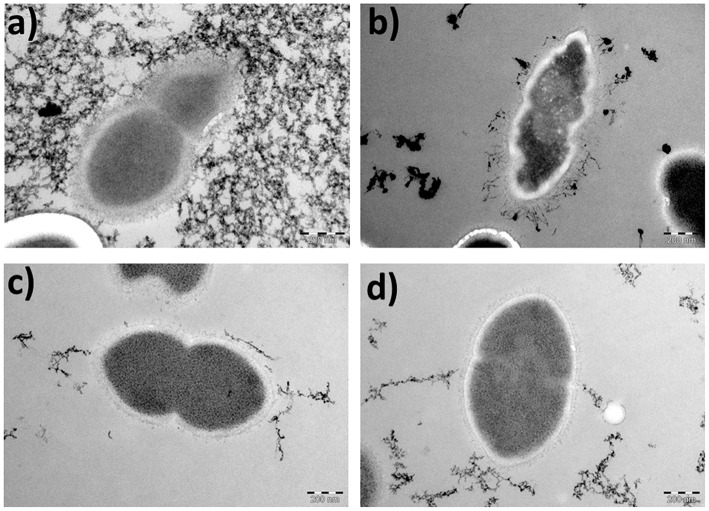
Transmission electron micrographs showing the morphological appearance of *S. pneumoniae* strain 110.58 and its ΔORF 1 mutant in the presence (0.07 mg/ml) and absence of ORF 1 peptide (magnification, × 53,000). **(a)** 110.58 control, **(b)** 110.58 + ORF 1 peptide, **(c)** ΔORF1 mutant control, and **(d)** ΔORF1 mutant + ORF 1 peptide (Background staining is artifactual).

To assess further the effect of the ORF 1 peptide on pneumococcal cell death, strain 110.58 and its mutant were incubated with the ORF 1 ligand for 30 min, harvested and examined by TEM. In the absence of the ORF 1 peptide, *S. pneumoniae* strain 110.58 cells had a normal diplococcal appearance (Figure 4a). In the presence of the ORF 1 peptide, some cells appeared to have apoptosis-like morphology: disrupted structures and shrinkage with loss of coccal shape (Figure 4b). Additional micrographs are shown in Figure S4 where “ghost cells” with cleared cytoplasm can be seen. The ΔORF 1 mutant cells in the presence and absence of the ORF 1 peptide appeared to have a typical pneumococcal appearance (Figures 4c,d).

## Discussion

Non-encapsulated pneumococci are considered to be less virulent than encapsulated strains but are sometimes isolated from sterile sites and make up more than 10% of nasopharyngeal isolates (Finland and Barnes, 1977; Carvalho et al., 2003). Current vaccines offer no protection against non-encapsulated strains and this may be selecting for their increased prevalence (Sá-Leão et al., 2009).

Non-encapsulated *S. pneumoniae* of multilocus sequence type 344, such as Swiss strain 110.58, have interesting features including increased colonization ability and transformation rate compared to encapsulated strains (Hathaway et al., 2004; Hanage et al., 2006; Hilty et al., 2014). They have two genes, *aliB-*like ORF 1 and *aliB-*like ORF 2, in place of the capsule genes, that are homologs of the gene encoding AliB substrate binding protein, found in all pneumococci (Hathaway et al., 2004). Previously, we have proposed a mechanism of interspecies communication consisting of specific binding of peptides derived from other bacterial species found in the nasopharyngeal microbiota via such substrate binding proteins, triggering changes in pneumococcal phenotype (Hathaway et al., 2014; Nasher et al., 2018a,b,c). AliB-like ORF 1 binds specifically to peptide SETTFGRDFN, matching 50S ribosomal subunit protein L4 of Enterobacteriaceae, promoting competence for genetic transformation in the presence of CSP (Hathaway et al., 2014). Exposure to *E. coli*, not a usual nasopharyngeal resident, which possesses the peptide, specifically reduced the number of live AliB-like ORF 1-expressing pneumococci. Here, the aim was to investigate how binding of AliB-like ORF 1 peptide affects pneumococcal phenotype. Our main conclusion is that specific binding of ORF 1 peptide to its receptor, as would occur in a stressful environment, causes *S. pneumoniae* to respond by undergoing apoptosis in a proportion of the population. We speculate that this is a mechanism to promote transmission of the remaining living pneumococci.

We were led to this conclusion by the following: The transcriptome profile of the wild type strain obtained by RNA sequencing showed upregulation of *lytFN1*, a gene encoding a caspase superfamily domain protein; peptidase C14 following exposure to the peptide. This was confirmed by real-time RT-PCR. In prokaryotes, caspase homologs are dubbed metacaspases (Asplund-Samuelsson et al., 2012), and have been reported to play a role in programmed cell death (PCD) in some bacteria including *Xanthomonas campestris* and *Trichodesmium erythraeum* (Berman-Frank et al., 2004; Wadhawan et al., 2010). Metacaspases may be cotranscribed with genes involved in hydrolysis, carbon metabolism and signaling processes (Asplund-Samuelsson et al., 2012). This is interesting because the adjacent gene, *lytFN2*, encoding an N-acetylmuramoyl-L-alanine amidase family protein was also upregulated in our RNA-Seq and real-time RT-PCR data following exposure to the peptide in the wild type. The product of this gene is a peptidoglycan hydrolase (Vollmer et al., 2008): a group of proteins associated with fratricide and the development of autolysis. Peptidoglycan hydrolases cleave elements of bacterial cell wall and *Staphylococcus aureus* amidases have been shown to have a role in PCD (Rice and Bayles, 2003). PCD has been reported to occur as a consequence of stress in *Xanthomonas* species (Chaloupka and Vinter, 1996; Berman-Frank et al., 2004; Wadhawan et al., 2010), as a defense mechanism during phage infections (Hazan et al., 2004) and during antibiotic treatment in *E. coli* (Chaloupka and Vinter, 1996). The role of peptidoglycan hydrolases in autolysis and fratricide supports our hypothesis that *lytFN1* and *lytFN2* may play a combined role in PCD in response to the AliB-like ORF 1 peptide ligand. LytFN1 and LytFN2 did not appear in the significantly upregulated proteins which we speculate is due to the timepoint being too early for their transcripts to be translated into protein.

Although we have not shown directly that LytFN1 and LytFN2 cause pneumococcal death in response to ORF 1 peptide, their predicted functions led us to our principal finding that ORF 1 peptide increases cell death specifically when bound by AliB-like ORF 1. When taken up by another route, as in the ΔORF 1 mutant, cell death is not observed but rather the bacteria use the peptide to boost growth. *LytFN1* and *lytFN2* are present at about 7% frequency in the pneumococcal population in both encapsulated and nonencapsulated pneumococci (Croucher et al., 2015). However, the link between PCD and ORF 1 peptide would only be seen in pneumococci which possess AliB-like ORF 1 and we do not exclude that these genes could also be involved in fratricide since they share homology with other cell wall hydrolases.

Binding of ORF 1 peptide to its receptor has been proposed to trigger a stress response in the pneumococcus (Hathaway et al., 2014) and we did find upregulation of the stress-related protein PadR-like family transcription regulator protein and transcription of *dpnM* associated with competence for genetic transformation (Johnston et al., 2013). The pneumococcus has been reported to undergo competence for genetic transformation during stressful conditions (Engelmoer and Rozen, 2011), although we did not see upregulation of other competence genes. In our previous study (Hathaway et al., 2014) we reported an increase in competence for genetic transformation in the presence of the ORF 1 peptide ligand together with CSP.

The regulatory link between metabolism and PCD has been reported to be widespread in nature (van den Esker et al., 2017). *ccpA* gene, encoding catabolite control protein A, was upregulated in response to ORF 1 peptide. CcpA is a transcriptional regulator that affects the expression of over 15% of the pneumococcal genome, including genes involved in virulence, regulation and central metabolism (Görke and Stülke, 2011). Regulating genes that are used to catabolize a specific carbon source until the cell has exhausted the favored source is a strategy that allows for maximum fitness, and is usually achieved by regulation of genes through global regulators (Carvalho et al., 2011; Görke and Stülke, 2011). We also noticed changes in expression of other metabolism-associated proteins in response to the ORF 1 peptide. Although the association between *ccpA* and the metabolism-associated genes shown here to be affected by the ORF 1 peptide has not been reported, it is plausible that this transcriptional regulator might have an indirect effect on their regulation. It is worth noting that in the experiments performed here *in vitro* we used CDM supplemented with glucose and studied planktonic growth. *In vivo* during nasopharyngeal colonization the pneumococci would be expected to form biofilm and to use galactose as their principal carbon source (Blanchette et al., 2016). Therefore, we cannot rule out that the effect of ORF 1 peptide on pneumococci in their natural environment would differ from that reported here.

ORF 1 peptide caused death of a proportion of the pneumococcal population but only when bound by the AliB-like ORF 1 receptor. The number of dead cells was equivalent to the number of additional live bacteria produced in response to the peptide meaning that the population of live bacteria remained stable. In order to study the cell death further we looked at the phenotype of the bacteria, following exposure to the peptide, by electron microscopy. Death of pneumococci by autolysis during stationary phase has been described to be due to the production of hydrogen peroxide by SpxB resulting in a death process exhibiting features of apoptosis (Regev-Yochay et al., 2007). These features were increased annexin V staining, decreased DNA content and apoptotic appearance by electron microscopy. Here, following exposure of wild type cells to ORF 1 peptide, we also saw features of apoptosis by electron microscopy despite the fact that our bacteria were in exponential phase rather that stationary phase. We observed shrinkage with loss of coccal shape and disruption of internal structures after 30 min of incubation of wild type pneumococci with peptide similar to the phenotype that Regev-Yochay et al. (2007) saw six hours into stationary phase. After only 30 min of exposure to peptide some pneumococci already had the phenotype of “ghost cells”. Ghost cells were described by Regev-Yochay et al. (2007) at twelve hours of stationary phase as having clearly disrupted structures, loss of coccal shape and clearing of the cytoplasm. In our experiments the appearance of the bacteria by electron microscopy and the activation of the caspase and amidase genes lead us to speculate that the cells are undergoing PCD.

We predict that pneumococci undergoing PCD would release pneumolysin, thereby increasing transmission of the remaining live population to a new host and thus escaping from the hostile environment. The mechanism of pneumolysin-mediated transmission has been reported in an *in vivo* mouse model (Zafar et al., 2017). Although AliB-like ORF 1 may bind other peptides from the environment and the effect could be different from that reported here, it would be interesting to determine the effect of its peptide ligand on transmission *in vivo*. The relevance of this phenotype is linked to the universal prevalence of AliB-like ORF 1 in the classic lineage of non-encapsulated pneumococci, a population with high non-susceptibility rates to β-lactams and other antimicrobials (Hilty et al., 2014). AliB-like ORF 1 ligand is found in the 50S ribosomal subunit protein L4 of many *Enterobacteriaceae* species including *Klebsiella pneumoniae*, a respiratory pathogen which may occupy the same niche as *S. pneumoniae*, and *E. coli* (Hathaway et al., 2014). Proteobacteria have been found to increase in the respiratory tract during dysbiosis, for example during asthma (Hilty et al., 2010).

In summary, we did see a link between the pneumococcal transcriptome, proteome, and phenotype in response to the ORF 1 peptide. We propose that uptake of ORF 1 peptide by a route other than by the specific AliB-like ORF 1 receptor stimulates metabolism and growth, as seen in the ΔORF 1 mutant. However, uptake via the AliB-like ORF 1 receptor in the 110.58 wild type strain leads to expression of genes linked to autolysis, proteins associated with a stress response and ultimately cell death.

## Data Availability

The datasets supporting the conclusions of this article are included within the article and its additional files. Transcriptomic data has been deposited into NCBI GEO (Accession number: E-MTAB-8289).

## Author Contributions

LH conceived the study and edited the manuscript. FN drafted the manuscript and prepared samples for RNA-Seq and proteomics, performed growth and competition assays, live/dead assay and real-time PCR. FN and LH participated in design of the study. MK did all computational analyses concerning RNA-Seq data. MH processed the proteomics data and performed the label-free protein quantification. All authors were involved in data interpretation and gave final approval for publication.

### Conflict of Interest Statement

The authors declare that the research was conducted in the absence of any commercial or financial relationships that could be construed as a potential conflict of interest.
