# Supplementary material for: Peptide Occurring in Enterobacteriaceae Triggers Streptococcus pneumoniae Cell Death
Source: Front Cell Infect Microbiol. 2019 Sep 10;9:320. doi: 10.3389/fcimb.2019.00320 (PMC6748166; doi:10.3389/fcimb.2019.00320)
Supplement: Supplementary file 8 [file Data_Sheet_1.PDF]

Supplementary Material

Peptide occurring in Enterobacteriaceae triggers *Streptococcus pneumoniae* cell death

Fauzy Nasher[1,2], Min Jung Kwun[3], Nicholas J Croucher[3], Manfred Heller[4], Lucy J. Hathaway[1] *

[1] Institute for Infectious Diseases, Faculty of Medicine, University of Bern, Friedbühlstrasse 51, CH-3001 Bern, Switzerland

[2] Graduate School for Cellular and Biomedical Sciences, University of Bern, Bern, Switzerland.

[3] Department of Infectious Disease Epidemiology, Imperial College London, London W2 1PG, United Kingdom

[4] Proteomics and Mass Spectrometry Core Facility, Department for BioMedical Research (DBMR), University of Bern, Bern, Switzerland

*Corresponding Author:

Lucy J Hathaway

lucy.hathaway@ifik.unibe.ch

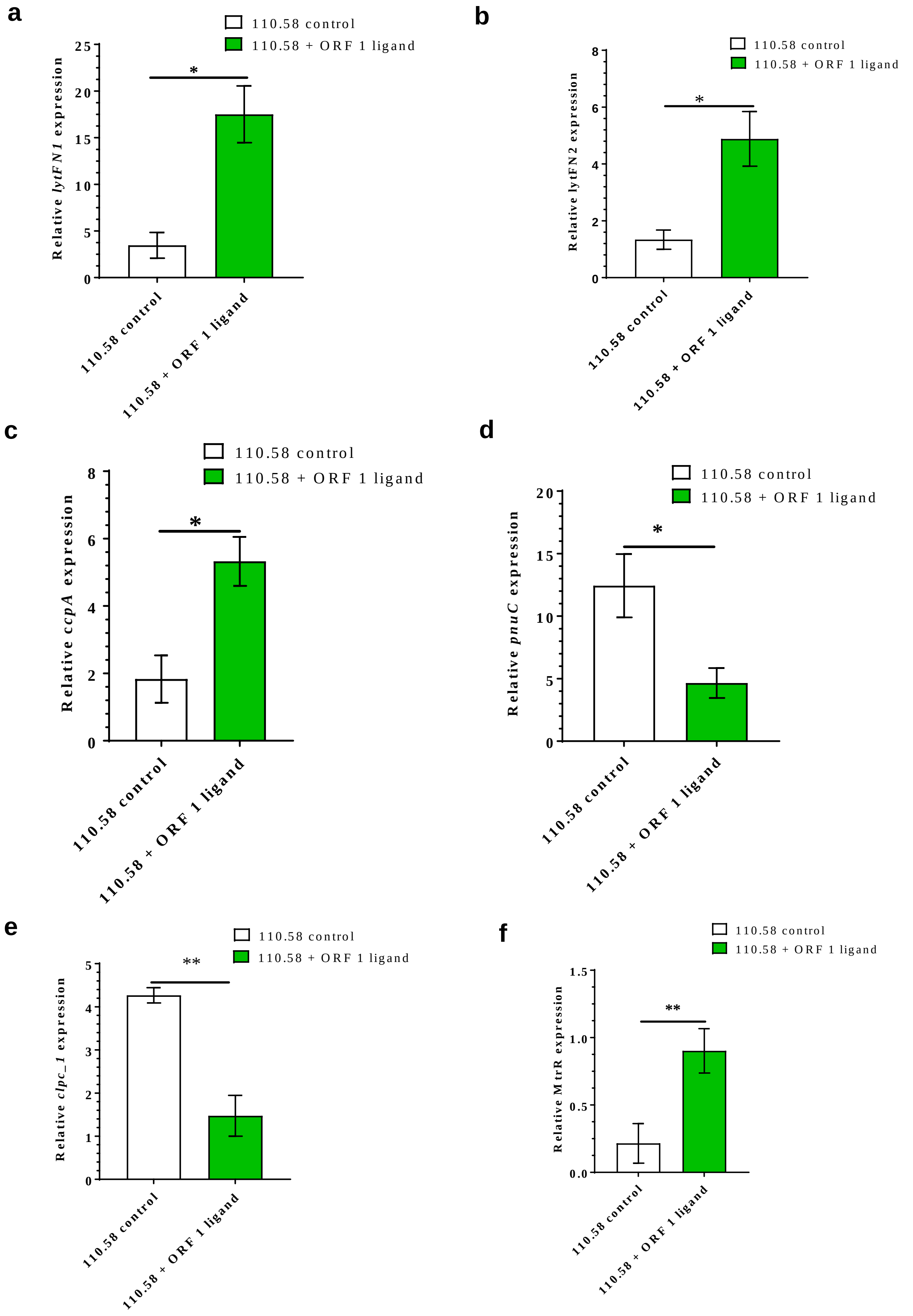
a
110.58 control
110.58 + ORF 1 ligand
Relative *lytFN1* expression
0
5
10
15
20
25
*
110.58 control
110.58 + ORF 1 ligand
b
110.58 control
110.58 + ORF 1 ligand
Relative lytFN2 expression
0
2
4
6
8
*
110.58 control
110.58 + ORF 1 ligand
c
110.58 control
110.58 + ORF 1 ligand
Relative *ccpA* expression
0
2
4
6
8
*
110.58 control
110.58 + ORF 1 ligand
d
110.58 control
110.58 + ORF 1 ligand
Relative *pnuC* expression
0
5
10
15
20
*
110.58 control
110.58 + ORF 1 ligand
e
110.58 control
110.58 + ORF 1 ligand
Relative *clpc_1* expression
0
1
2
3
4
5
**
110.58 control
110.58 + ORF 1 ligand
f
110.58 control
110.58 + ORF 1 ligand
Relative MtrR expression
0.0
0.5
1.0
1.5
**
110.58 control
110.58 + ORF 1 ligand

Figure S1 - Relative gene expression. a *lytFN1*, **b** *lytFN2*, **c** *ccpA_1*, **d** *pnuC*, **e** *clpC_1* and **f** MtrR expression was determined by real-time RT-PCR and is displayed relative to the value of the lowest expression, after normalization using 16S RNA gene expression. The values are the means of three separate experiments. Error bars indicate standard error, $^{*}p \leq 0.05$, $^{**}p \leq 0.01$. To verify the RNA-Seq results, we performed real-time RT-PCR on several genes (Figure 1). RT-PCR results were in line with the RNA-seq; in response to the ORF 1 peptide, *lytFN1* was upregulated 5.9 fold while *lytFN2* was upregulated 3.8 fold in the RNA-Seq results, both genes were upregulated 3 fold in the RT-PCR (Figure S1a and b). ccpA_1 was also upregulated by 1.6 fold in the RNA-Seq results and 3 fold in the RT-PCR in response to the ORF 1 peptide (Fig. S1c). *pnuC* was significantly downregulated 3 folds in response to the peptide (Figure S1d). RT-PCR also indicated clpC_1 was downregulated 2 folds in response to the peptide (Fig. S1e). In the proteomic profile, MtrR was significantly upregulated, RT-PCR results also showed a significant increase of gene expression of the gene encoding MtrR (Fig. S1f).

a)

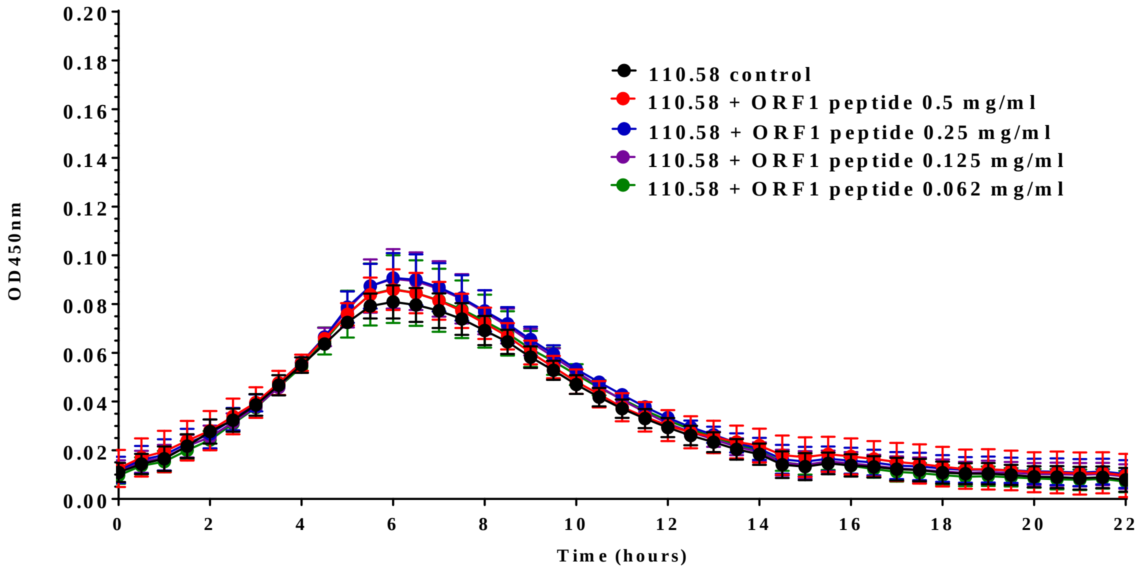

b)

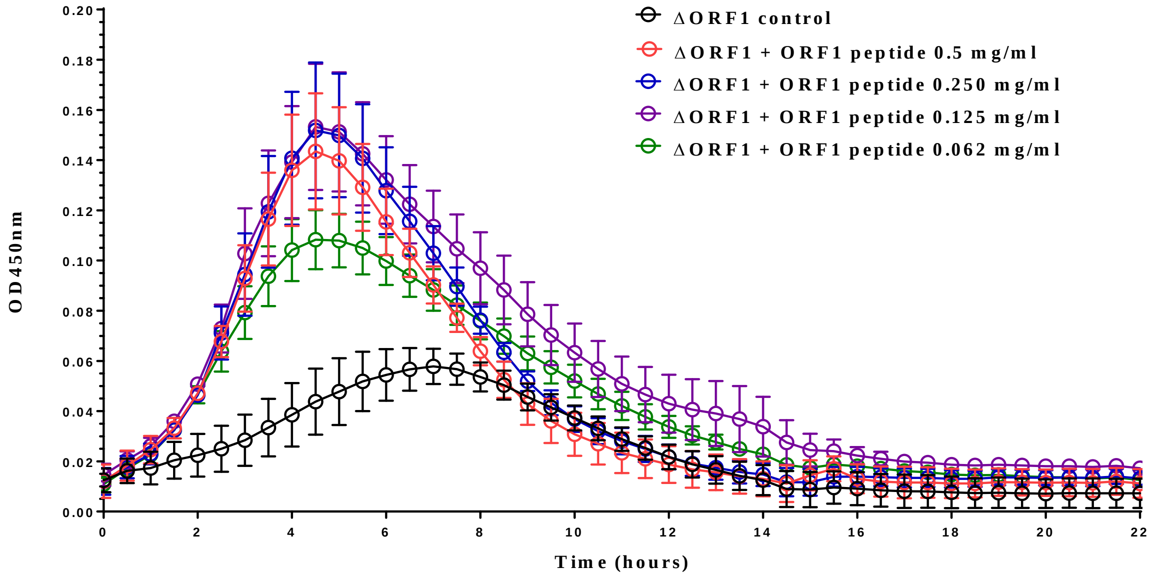

Supplementary Figure S2 – Growth of wildtype strain a) 110.58 and b) mutant ΔORF 1 in CDM with and without ORF1 peptide SETTFGRDFN at the concentrations indicated. Curves show the mean values for three independent experiments, error bars indicated SEM

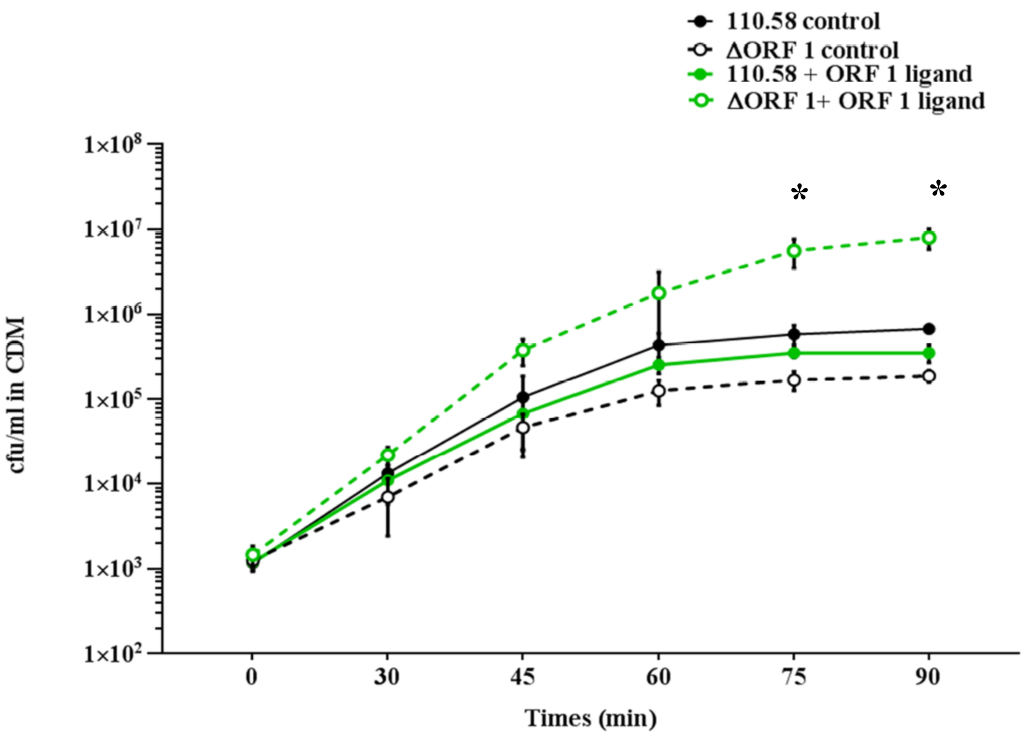

Supplementary Figure S3 - Growth analysis of pneumococcal strain 110.58 and its mutant Δ ORF 1 in the presence and absence of 0.062 mg/ml of ORF 1 peptide. The effect of the ORF 1 peptide on the number of live bacteria quantified by cfu counts. Error Bars indicate standard deviation, *$p \leq 0.05$.

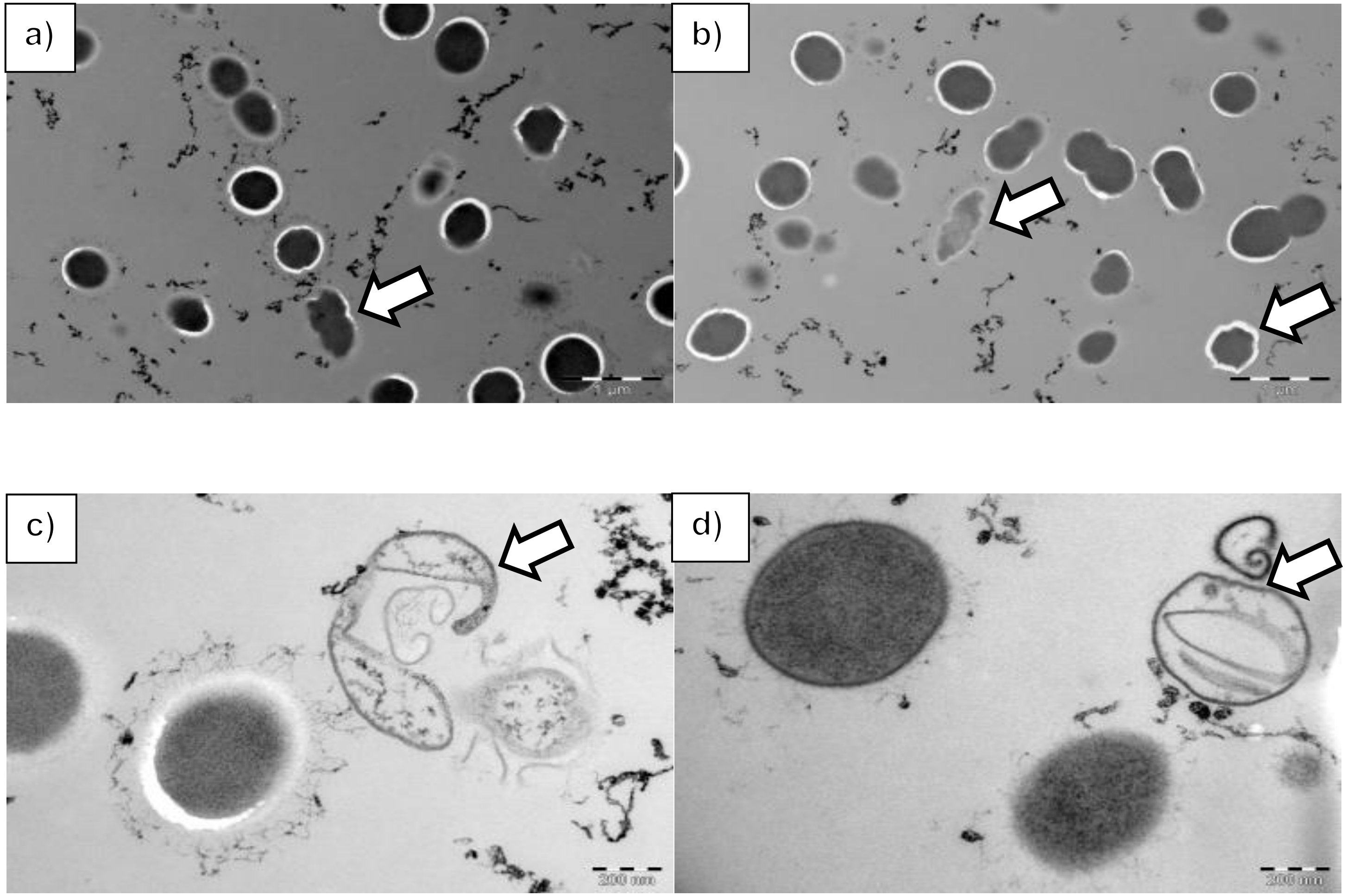

Supplementary Figure S4 – Transmission electron micrographs showing the morphological appearance of *S. pneumoniae* strain 110.58 in the presence of 0.07 mg/ml ORF 1 peptide. In a) and b) bars indicate 1 µm and arrows indicate cells undergoing shrinkage and loss of coccal shape. In c) and d) bars indicate 200 nm and arrows indicate ghost cells.
